# Ameliorative Effects by Hexagonal Boron Nitride Nanoparticles against Beta Amyloid Induced Neurotoxicity

**DOI:** 10.3390/nano12152690

**Published:** 2022-08-05

**Authors:** Nursah Aydin, Hasan Turkez, Ozlem Ozdemir Tozlu, Mehmet Enes Arslan, Mehmet Yavuz, Erdal Sonmez, Ozgur Fırat Ozpolat, Ivana Cacciatore, Antonio Di Stefano, Adil Mardinoglu

**Affiliations:** 1 Department of Molecular Biology and Genetics, Erzurum Technical University, Erzurum 25050, Turkey; 2 Department of Medical Biology, Faculty of Medicine, Atatürk University, Erzurum 25240, Turkey; 3 East Anatolia High Technology Application and Research Center (DAYTAM), Ataturk University, Erzurum 25240, Turkey; 4 REEM Neuropsychiatry Clinics, İstanbul 34245, Turkey; 5 Department of Nanoscience and Nanoengineering, Graduate School of Natural and Applied Sciences, Ataturk University, Erzurum 25240, Turkey; 6 Department of Physics, Kazım Karabekir Education Faculty, Atatürk University, Erzurum 25240, Turkey; 7 Computer Sciences Research and Application Center, Atatürk University, Erzurum 25240, Turkey; 8 Department of Pharmacy, University “G. d’Annunzio” of Chieti-Pescara, Via dei Vestini, 31, 66100 Chieti Scalo, CH, Italy; 9 Science for Life Laboratory, KTH-Royal Institute of Technology, SE-17121 Stockholm, Sweden; 10 Centre for Host-Microbiome Interactions, Faculty of Dentistry, Oral & Craniofacial Sciences, King’s College London, London SE1 9RT, UK

**Keywords:** Alzheimer’s disease, beta amyloid, hexagonal boron nitride nanoparticles, neurotoxicity, neuroprotection, SHSY5Y cells, in vitro

## Abstract

Alzheimer’s disease (AD) is considered as the most common neurodegenerative disease. Extracellular amyloid beta (Aβ) deposition is a hallmark of AD. The options based on degradation and clearance of Aβ are preferred as promising therapeutic strategies for AD. Interestingly, recent findings indicate that boron nanoparticles not only act as a carrier but also play key roles in mediating biological effects. In the present study, the aim was to investigate the effects of different concentrations (0–500 mg/L) of hexagonal boron nitride nanoparticles (hBN-NPs) against neurotoxicity by beta amyloid (Aβ_1-42_) in differentiated human SH-SY5Y neuroblastoma cell cultures for the first time. The synthesized hBN-NPs were characterized by X-ray diffraction (XRD) measurements, scanning electron microscopy (SEM) and transmission electron microscopy (TEM). Aβ_1-42_-induced neurotoxicity and therapeutic potential by hBN-NPs were assessed on differentiated SH-SY5Y cells using MTT and LDH release assays. Levels of total antioxidant capacity (TAC) and total oxidant status (TOS), expression levels of genes associated with AD and cellular morphologies were examined. The exposure to Aβ_1-42_ significantly decreased the rates of viable cells which was accompanied by elevated TOS level. Aβ_1-42_ induced both apoptotic and necrotic cell death. Aβ exposure led to significant increases in expression levels of APOE, BACE 1, EGFR, NCTSN and TNF-α genes and significant decreases in expression levels of ADAM 10, APH1A, BDNF, PSEN1 and PSENEN genes (*p* < 0.05). All the Aβ_1-42_-induced neurotoxic insults were inhibited by the applications with hBN-NPs. hBN-NPs also suppressed the remarkable elevation in the signal for Aβ following exposure to Aβ_1-42_ for 48 h. Our results indicated that hBN-NPs could significantly prevent the neurotoxic damages by Aβ. Thus, hBN-NPs could be a novel and promising anti-AD agent for effective drug development, bio-nano imaging or drug delivery strategies.

## 1. Introduction

Alzheimer’s disease (AD), which is considered the most common age-related neurodegenerative disorder, was first described in 1901 by the German psychiatrist Alois Alzheimer in his study of changes in brain tissue after the death of a patient with multiple neurological symptoms that followed for many years [1,2]. Although this disease was identified over a century ago, no descriptive etiology and no viable treatment have been found [3,4,5]. It is thought that the number of people affected by the disease will increase significantly due to better living conditions, longer lifetimes, and the absence of a definitive cure for AD [6,7]. Because of this, AD is a concern today and has become an urgent research priority [6].

AD is responsible for the majority of dementia cases, and it is characterized by significant memory loss, restrictions in daily life activities and various neuropsychiatric and behavioral disorders [8,9]. AD shows characteristic histopathological features such as intracellular neurofibrillary lump formation and extracellular amyloid accumulation in the brain [1]. Amyloid beta (Aβ) is the main factor in the formation of amyloid plaques is formed by proteolytic cleavage with β and γ secretases of amyloid precursor protein (APP), which is a type 1 transmembrane protein in the brain membrane. As a result of this division, several types of Aβ such as Aβ_1-40_ and Aβ_1-42_ are obtained [10,11]. Aβ_1-42_ tends to bind to the intracellular and extracellular layers of Aβ aggregates, which play a key role in the pathology of AD [12]. Great efforts are being directed towards designing and developing novel agents that act as Aβ fibril inhibitors for the prevention and treatment of AD [13,14,15,16].

Boron (B) exerts metal and non-metal properties as a semiconductor element. B is never found on Earth in the elementary form. B is present in nature in the form of borates like boric acid, borax, colemanite or ulexite [17]. Recent studies indicated positive health impacts due to B intake on humans and animals. These key biological effects include antioxidant [18], anti-mutagenic [19], anti-microbial [20], anti-inflammatory [21], anticancer [22], neuroprotective [23] action potential and metal chelating features [24].

BN is a synthetic compound consisting equal numbers of B and nitrogen (N) atoms in [25]. The layered lattice structure of BN forms the basis of many properties such as good lubricating properties, good insulating properties, high resistance to chemical attacks, high dielectric distortion, high volume resistance, high neutron capture capacity, excellent thermoelectric properties and good resistance to oxidation. BN is very similar to carbon structures, and it has attracted much attention due to its unique chemical, physical, thermal, mechanical, and biocompatibility properties. Hence, BN is widely used in a wide range of industrial fields due to its physico-chemical features and lack of toxicity [26,27,28]. Depending on the pressure and temperature, the BN molecule has different crystal structures such as hexagonal (h-BN), wurstitic (w-BN), rhombus (r-BN) and cubic (c-BN). However, the most stable form at room temperature is the hexagonal form [29,30]. Since h-BN has the same number of electrons as two carbon atoms, it is similar to the graphite structure. Due to this similarity, hBN is called “white graphite” or “white carbon” [31]. This material, which is structurally similar to graphene, has attracted much attention in recent years as it has been used to create different nanostructures such as BN nanotubes (BNNT) and BN nanosheets (BNNS) [30,32].

Boron nanoparticles (BNPs) drew considerable interest in a multitude of applications including high energy density fuels, hydrogen generation from water as well as neutron capture therapy of cancer cells [33]. Recent toxicogenomics studies of different BNPs including titanium diboride, zinc borate [34], tungsten boride [35], nickel boride [36] and boron carbide [37] showed that BNPs could be used as safe nanomaterials in several pharmacological and biomedical applications. Considering the applications of BN in the field of nanotechnology, studies on the production of BN in nanostructure and determination of its properties have gained importance. Nano BN products exert superior properties such as being lighter, more elastic and harder than existing BN products in powder form [38]. Similar to other BNPs, BNNTs do not have toxic effects on kidney cells, and thus, they are considered as biocompatible materials [28]. Interestingly, BN nanoparticles are enabled to positively alter cell cycle, signal transduction, cell–cell interactions and cancer affecting genes in human lung epithelial cells [39]. In addition, BN nanoparticles exert low cytotoxicity potential, high chemical stability, high drug loading efficiency and rapid cell uptake [40].

In this investigation, we have attempted to establish Aβ neurotoxicity by exposing differentiated human SHSY5Y cells to Aβ_1-42_. We synthesized hBN-NPs and morphologically verified them using XRD, SEM and TEM analysis methods. Then, we investigated their role in ameliorating the neurotoxicity induced by Aβ_1-42_ using an AD-like in vitro model. Our results propound the role of hBN-NPs in modulating neurotoxic damage by Aβ_1-42_. Our proposed insight could be fruitful and crucial in developing novel and multitargeted treatment options or drug delivery strategies towards AD.

## 2. Materials and Methods

### 2.1. Synthesis of hBN-NPs

In the synthesis of hBN-NPs, boric acid (H_3_BO_3_), sodium azide (NaN_3_) and hydrazine hydrate (N_2_H_4_·H_2_O) chemicals were used as B and N sources. First, 2.5 g of H_3_BO_3_ and 8 g of NaN_3_ chemicals were dissolved in 300 mL of deionized water, and mixing was carried out for 30 min with a magnetic stirrer. Then, after adding 2.3 mL of N_2_H_4_·H_2_O to the solution, the solution was stirred for a further 30 min. After mixing, the solution was placed in an autoclave in nitrogenous gas medium and kept in the autoclave ash oven at 300 °C for 16 h. After 16 h, the product removed from the autoclave was washed with plenty of water. The product was then subjected to deionized water drying at 100 °C for 2–3 h in a vacuum environment and hBN-NPs were synthesized [41,42].

### 2.2. Characterization of hBN-NPs

The crystal structure of the synthesized hBN-NPs was characterized by XRD measurements, while the particle shape, size distribution and elemental composition were evaluated using scanning electron microscopy (SEM) and transmission electron microscopy (TEM). In this study, Rigaku SmartLAb X ray diffractometer was used in hBN XRD analysis. SEM images of the samples were obtained with Jeo Jsm-6610 brand SEM device, TEM images were obtained with Philips CM30T brand TEM device.

### 2.3. Cell Cultures

The human neuroblastoma SH-SY5Y cells from ATCC (CRL-2266) were cultured in Dulbecco’s modified Eagle medium (DMEM): F12 (Gibco, New York, NY, USA) supplemented with 10% fetal bovine serum (FBS) (Gibco), 1% penicillin and streptomycin at 37 °C in a 5% CO_2_. Cells were seeded onto plates and were passaged when they reached 70–80% confluence. For the differentiation of SH-SY5Y cells, the medium was replaced with DMEM: F12 medium containing 1% FBS and 10 μM retinoic acid (RA) (Sigma-Aldrich, Milan, Italy). The media of the cells were renewed every three days with 1% FBS and 10 μM RA containing medium. The differentiation process of the cells was observed for 11 days with light microscopy [43].

### 2.4. Cell Viability Testing

#### 2.4.1. MTT Assay

Cell viability was measured by using MTT Cell Proliferation Assay Kit (Cayman Chemical, Ann Arbor, MI, USA) according to the manufacturer’s manual. Briefly, 1 × 10^4^–1 × 10^5^ cells were seeded in 96-well plates and kept under appropriate culture conditions (37 °C, 5% CO_2_) for 24 h for cell attachment. Stock Aβ peptides were incubated in DMEM: F12 medium for 24 h at 37 °C to activate the fibrilization to get toxic form of peptides. Then, cells were incubated with both Aβ (10 mM, Human, Sigma-Aldrich, Milan, Italy) and different concentrations (0–500 mg/L) of hBN-NPs for 48 h. Then, MTT solution was added to the cell cultures and incubated for 3 h in 37 °C, 5% CO_2_. Formazan crystals were then dissolved in dimethyl sulfoxide (DMSO). The absorbance of each sample was measured at 570 nm in a microplate reader (Synergy-HT; BioTek, Winooski, VT, USA). As a positive control group, cells were treated with 1% (*v*/*v*) Triton X-100 [44].

#### 2.4.2. LDH Assay

LDH level was measured by using LDH Cytotoxicity Assay Kit (Cell Biolabs, Ann Arbor, MI, USA) according to the manufacturer’s manual. Briefly, 1 × 10^4^–1 × 10^5^ cells were seeded in 96-well plates and kept under appropriate culture conditions (37 °C, 5% CO_2_) for 24 h for cell attachment. Then, cells were incubated with both Ab (10 μM) and different concentrations (0–500 mg/L) of hBN-NPs for 48 h. After incubation, 90 µL was taken from the wells and transferred to the new 96-well plate. Then, LDH reagent was transferred to each of the 96 wells and incubated for 30 min in the dark at room temperature. Finally, the absorbance of the samples was read at 450 nm using a microplate reader [44].

#### 2.4.3. Assays for Oxidative Stress and Antioxidant Status

TAC and TOS analyzes, known as automated and colorimetric methods, were measured using commercially available kits (Rel Assay Diagnostics, Gaziantep, Turkey) according to the provider’s instructions. Ascorbic acid (10 μM) and hydrogen peroxide (25 μM) from Sigma-Aldrich Company were preferred as positive control treatments in determining TAC and TOS levels, respectively [45].

#### 2.4.4. RNA Isolation

PureLink RNA Mini Kit (Invitrogen, Waltham, MA, USA) was used for RNA isolation and was performed according to the manufacturer’s instructions. Then, cDNA synthesis was conducted using 10 µL RNA with High-Capacity cDNA Reverse Transcription Kit (Applied Biosystems, Waltham, MA, USA) following to the provider’s manual. q-PCR was carried out using Sybr Green Master Mix (Applied Biosystems) on a Real-Time PCR Detection System (Qiagen Rotor-Gene Q). The qPCR program was: 50 °C for 2 min, 95 °C for 10 min × 40 cycles, 95 °C for 15 s, and 60 °C for 1 min. The primers used for RT-qPCR are listed in Supplementary Table S1. β-actin was used as a reference gene [46].

#### 2.4.5. Hoechst 33258 Fluorescent Staining

Hoechst 33258 staining was used to assess characteristic apoptotic morphological changes in the cells treated with borax. As previously described [47], treated cells were fixed with 4% paraformaldehyde for 30 min at 37 °C and stained with Hoechst 33258 (Sigma-Aldrich, Schnelldorf, Germany) for 15 min. Stained nuclei were then observed with a fluorescence microscope (Leica, DM IL LED) to determine nuclei fragmentation and chromatin condensation.

## 3. Statistical Analysis

The results obtained from the studies were analyzed using SPSS 20.0 program. Statistical evaluations were made using one-way analysis of variance (ANOVA) and Duncan’s test. Results are presented as mean ± standard error of six independent repetitions. A *p*-value of less than 0.05 was considered as statistically significant.

## 4. Results

**Figure 1:**
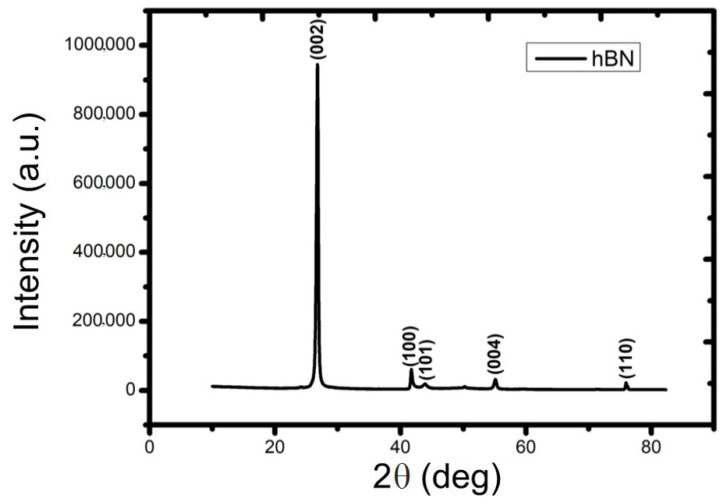
XRD pattern of hBN-NPs.

The crystal structure of the synthesized hBN-NPs was characterized by XRD measurements. The XRD results of hBN nanoparticles were shown in Figure 1. Five dominant peaks were observed on the XRD plot of hBN nanoparticles. The main peak obtained by XRD analysis of hBN-NPs corresponded to 2θ = 26.76° at the Bragg angle, while other peaks corresponded to 2θ = 41.70°, 43.91°, 55.12° and 75.97° [48]. The planar values of these peaks in the Müller index were found to correspond to 002, 100, 101, 004 and 110. All of the sharp and well-defined peaks observed in the XRD graph corresponded to hBN-NPs (JCPDS File 89-7102) and proved the hexagonal structure of BN.

**Figure 2:**
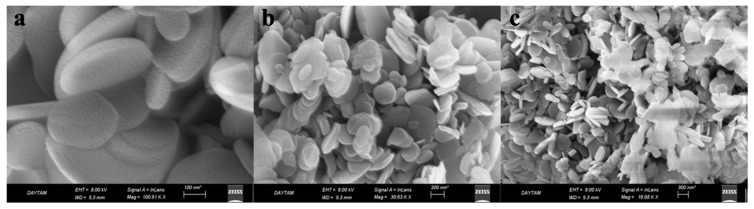
Images of hBN-NPs at different scales under the SEM: (**a**) 100 nm, (**b**) 200 nm, (**c**) 300 nm.

**Figure 3:**
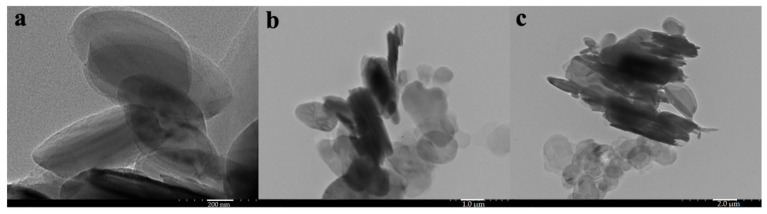
Images of hBN-NPs at different scales under the TEM: (**a**) 200 nm, (**b**) 1.0 µm, (**c**) 2.0 µm.

Particle shape, size distribution and elemental composition of the synthesized hBN-NPs were evaluated using imaging techniques including SEM and TEM microscopes. When looking at the morphology of hBN-NPs with SEM and TEM image analysis, it was seen that the nanoparticles had a platelet-like hemispherical or long shape. In addition, it was observed that the surfaces of hBN-NPs showed homogeneous properties. The synthesized hBN-NPs were determined to have sizes ranging from 100 to 300 nm. Images of hBN-NPs in different scales using SEM and TEM microscopy were presented in Figure 2 and Figure 3.

**Figure 4:**
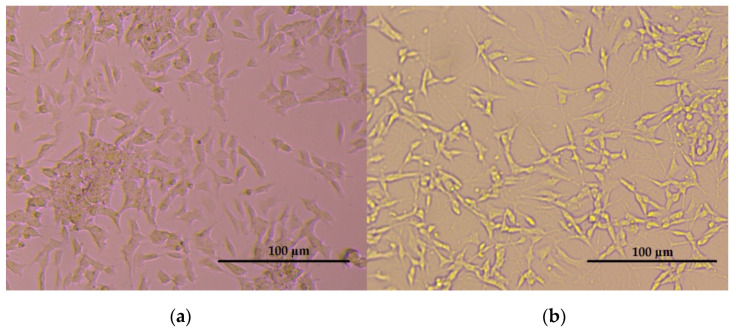
(**a**) Undifferentiated SH-SY5Y cells; (**b**) SH-SY5Y cells differentiated via treatment with RA.

**Figure 5:**
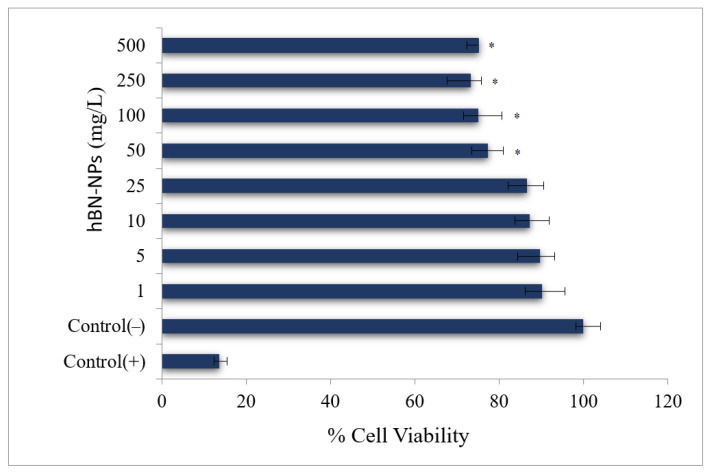
Cell viability (MTT assay) in differentiated SH-SY5Y cell cultures maintained 48 h in the presence of hBN-NPs. (Control (−): Cells without hBN-NPs; Control (+): Cell treated with Triton-X). * Symbol presents significant differences at the *p* < 0.05 level from the control group.

**Figure 6:**
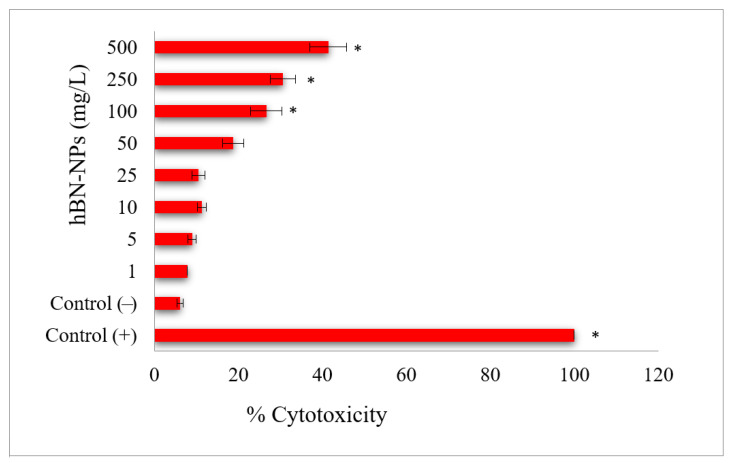
Cell viability (LDH assay) in differentiated SH-SY5Y cell cultures maintained 48 h in the presence of hBN-NPs. (Control (−): Cells without hBN-NPs; Control (+): Cell treated with Triton-X. * Symbol presents significant differences at the *p* < 0.05 level from the control group).

**Figure 7:**
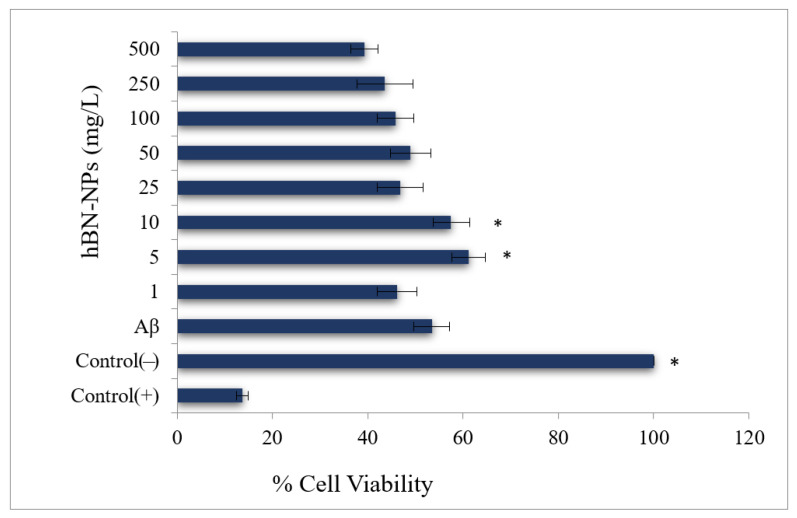
Effects of hBN-NPs on Aβ toxicity in cultured differentiated human SH-SY5Y cells as measured by mitochondrial activity (MTT assay). (Control (−): Cells without hBN-NPs; Control (+): Cell treated with Triton-X. * Symbol presents significant differences at the *p* < 0.05 level from the Aβ group).

**Figure 8:**
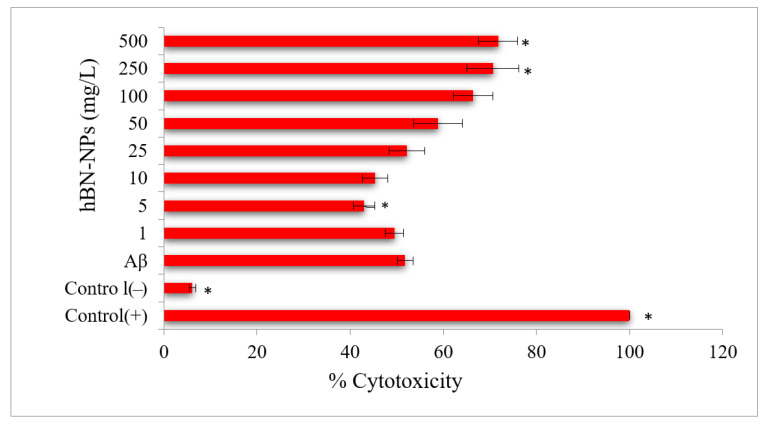
Effects of hBN-NPs on Aβ toxicity in cultured differentiated human SH-SY5Y cells as measured by LDH release assay. (Control (−): Cells without hBN-NPs; Control (+): Cell treated with Triton-X. * Symbol presents significant differences at the *p* < 0.05 level from the Aβ group).

Aβ-induced neurotoxicity potential and damage to membrane integrity were evaluated on differentiated SH-SY5Y cells (Figure 4) by MTT and LDH assays. Treatments with 1, 5, 10 and 25 mg/L of hBN-NPs did not alter the cell viability rates as compared to untreated cells in MTT assay (*p* > 0.05) (Figure 5). Likewise, 1, 5, 10, 25 and 50 mg/L of hBN-NPs did not lead to statistically significant elevations of LDH release when compared to untreated cells in LDH assay (*p* > 0.05) (Figure 6). Moreover, the highest concentration of hBN-NPs (10 mg/L) caused around 12% reduction of cell viability rates. In fact, 500 mg/L of hBN-NPs led to decreases of cell viability rates in the rates of 25.0% and 41.4%, in MTT and LDH release assays, respectively (Figure 5 and Figure 6). On the contrary, treatment of 50 μM of Aβ_1-42_ for 48 h nearly killed 50% of the total cell populations. In addition, we determined the ameliorative effect of hBN-NPs against Aβ_1-42_ induced neurotoxicity in differentiated SHSY5Y cells using MTT and LDH assays. According to the MTT cell viability assay, hBN-NPs were found to be more effective at the concentrations of 5 and 10 mg/L against Aβ_1-42_ (Figure 7 and Figure 8).

**Figure 9:**
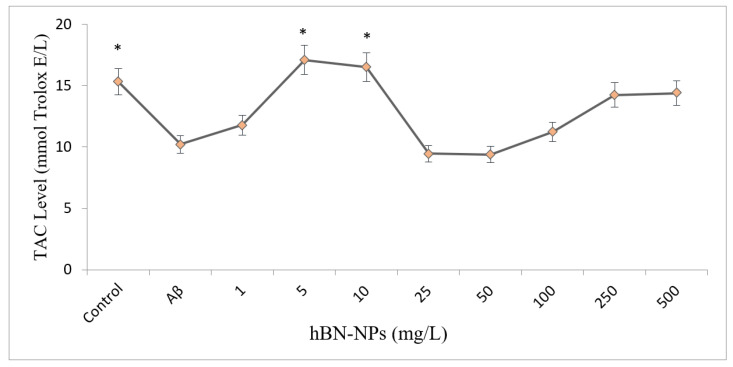
Effects of hBN-NPs and Aβ treatments on TAC levels in differentiated SH-SY5Y cells. (Control: Cells without hBN-NPs. * Symbol presents significant differences at the *p* < 0.05 level from the Aβ group).

**Figure 10:**
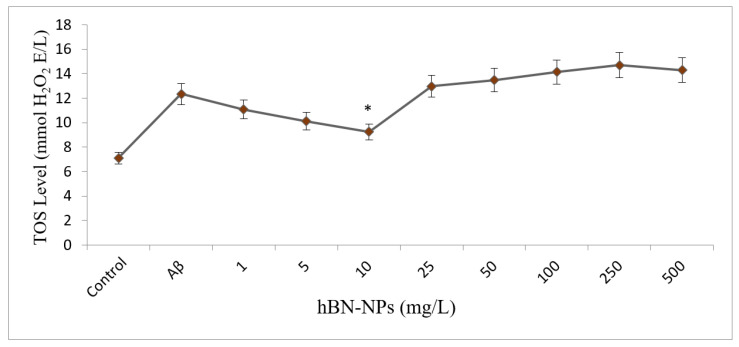
Effects of hBN-NPs and Aβ treatments on TOS levels in differentiated SH-SY5Y cells. (Control: Cells without hBN-NPs. * Symbol presents significant differences at the *p* < 0.05 level from the Aβ group).

TAC and TOS levels were carried out to understand the associated mechanism behind the neuroprotective action by hBN-NPs. The results showed that Aβ_1-42_ caused a statistically significant decrease in TAC level and a significant increase in TOS level when compared to control (*p* < 0.05). Moreover, when the cells treated with different concentrations of hBN-NPs against Aβ_1-42_, the measured TAC level increased from 10.21 ± 0.14 to 17.09 ± 0.18 mmol Trolox Equiv./L, and TOS levels decreased from 12.35 ± 1.64 to 9.25 ± 0.67 mmol H_2_O_2_ Equiv./l (Figure 9 and Figure 10).

**Figure 11:**
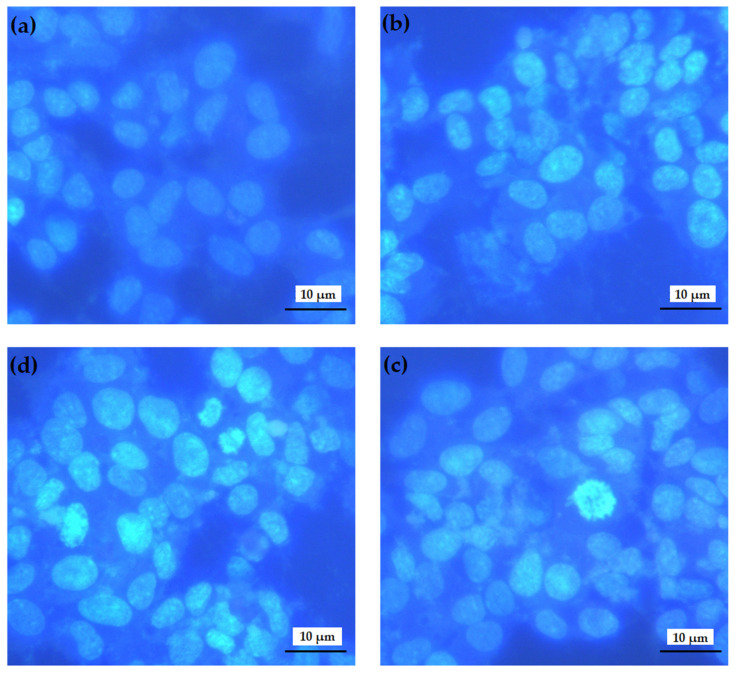
Effects of hBN-NPs and Aβ on morphological changes in differentiated SH-SY5Y cells: (**a**) untreated SH-SY5Y cells, (**b**) hBN-NPs (10 mg/L), (**c**) cell culture containing only Aβ, (**d**) hBN-NPs plus Aβ in 40× magnification.

Hoechst 33258 staining was used to analyze the chromosomal integrity of SH-SY5Y cells exposed to Aβ_1-42_ and hBN-NPs (Figure 11). Microscopic analysis showed that the Aβ_1-42_ application disrupted the nucleus of the cells and decreased the number of healthy cells. On the other hand, the applications with 10 mg/L of hBN-NPs ameliorated the toxic effect by Aβ_1-42_ and decreased the necrotic nuclei formations for 48 h.

**Figure 12:**
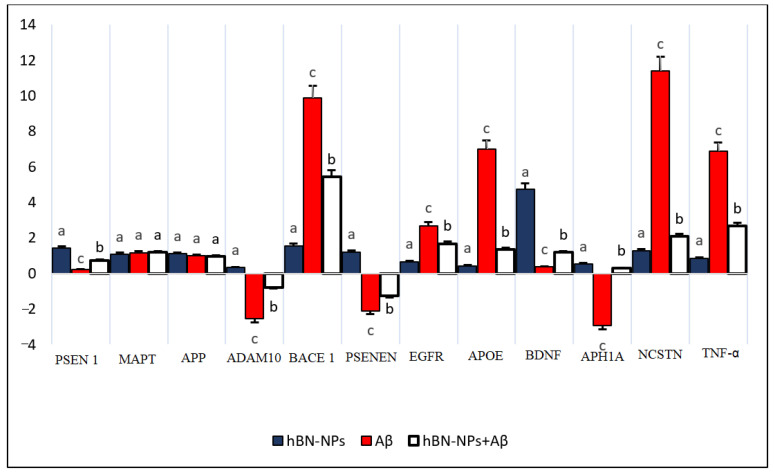
Effects of hBN-NPs and Aβ applications on expression of AD-related genes in differentiated SH-SY5Y cells. The mean ± SE values with different superscript letters on the bars for each gene are significantly different (*p* < 0.05).

In order to determine the molecular basis of the mechanism of action of hBN-NPs, the expression levels of 12 AD-related genes were investigated. Aβ_1-42_ exposure caused significant increases in expression levels in APOE, BACE 1, EGFR, NCTSN and TNF-α genes and significant reductions in the expression of ADAM 10, APH1A, BDNF, PSEN1 and PSENEN genes in the cells. BDNF expression was remarkably elevated after alone treatment with 10 mg/L of hBN-NPs. Importantly, the altered gene expression levels by Aβ_1-42_ were ameliorated after cotreatment with hBN-NPs and Aβ_1-42_. However, *APP* and *MAPT* expressions were not changed after treatment with hBN-NPs, Aβ_1-42_ or hBN-NPs plus Aβ_1-42_ as compared to untreated cultures (Figure 12).

## 5. Discussion

Nanotechnology is moving at a dizzying speed and promises great hopes, especially in the field of medicine [49,50]. Nanotechnology is thought to have the potential to revolutionize the treatment, diagnosis, monitoring and control of neurodegenerative diseases such as AD [51]. It has been concluded that the use of nanostructures will facilitate the development of tools necessary for the diagnosis and treatment of AD [10]. For these purposes, nanoparticles with unique physical, chemical, electronic, optical, magnetic and mechanical properties are widely used [51]. There have been few studies investigating the toxic effects of boron nanomaterials, which are of interest and have a large potential due to their superior properties [52,53]. In this study, the neuroprotective effect of hBN-NPs was investigated. Firstly, MTT and LDH analyzes were used to evaluate the cytotoxicity by hBN-NPs nanoparticles in differentiated SH-SY5Y cell cultures. According to results of cytotoxicity endpoints, the concentrations below than 50 mg/L of hBN-NPs were found non-cytotoxic for differentiated SH-SY5Y cell cultures. The hBN-NPs concentrations between 10 and 50 mg/L led to slight cytotoxic potential. Our findings are in line with the limited previous data recorded in the literature. It was observed that BN-NPs could affect the cells in time and concentration dependent manners [54]. In addition, it was found that there was no significant change in cellular morphology at low doses of BN nanostructures, but cellular stress could occur due to increasing concentrations. These results indicate that low dose BN-NPs can be used in biomedical applications safely [55]. The results of several studies also show that BNNTs exhibit good biocompatibility in sufficient concentrations for potential pharmacological applications, are a potential biomaterial for advanced biomedical uses, cyto-compatible and much safer than conventional nanomaterials [52,56,57].

Relatively low concentrations (<50 mg/L) of hBN-NPS provided significant cytoprotection against Aβ_1-42_ induced neurotoxicity in AD-like in vitro model. To determine whether the neuroprotective action by hBN-NPs was related to oxidative alterations, TAC and TOS levels were investigated. The treatments with hBN-NPs significantly ameliorated the alterations in TAC and TOS level induced by Aβ exposure. In fact, TAC level was significantly increased after hBN-NPs exposure, and there was also significant differences in TOS levels as compared to Aβ_1-42_ treated cell cultures. Numerous studies have shown that oxidative stress plays an important role in the pathogenesis of a number of diseases such as ischemia, cancer, diabetes, head trauma, Parkinson’s disease, amyotrophic lateral sclerosis, malaria, Down syndrome, Huntington’s disease and AD [56,57]. It was determined that neurons in the brain significantly oxidized ROS compared to other organs and as a result contributed to neuronal damage in aging and neurological diseases [57,58]. A number of studies have revealed that patients with AD showed high levels of oxidative stress, characterized by free radical formation, protein, DNA and RNA oxidation, elevated lipid peroxidation, mitochondrial dysfunction, and inactivation of antioxidant enzymes [58,59]. Oxidative stress, one of the earliest events in the pathogenesis of AD, was found to be effective in the progression of AD, causing overproduction of Aβ peptides, tau hyperphosphorylation, degeneration and death of neurons by β-secretase activation [59,60,61]. Oxidative damages and Aβ were linked to each other because Aβ generated oxidative stress in both in vivo and in vitro conditions. In parallel to our findings, Aβ was found to be responsible for increased free radical production in neurons, then leading to oxidative stress and cell death [62]. Considerable efforts were made on several antioxidants that were able to scavenge free radicals, thus providing protection against oxidative damages [63]. In this context, the neuroprotection by hBN-NPs against Aβ toxicity could be associated with their antioxidant features. As a matter of fact, hBN-NPs exerted antioxidative action against 1-methyl-4-phenylpyridinium (MPP+)-induced experimental Parkinson Disease model using differentiated pluripotent human embryonal Ntera-2 carcinoma cell cultures [64]. In similar to this in vitro study, hBN-NPs (100 µg/kg) supported in vivo antioxidant capacity without leading oxidative stress in serum samples of experimental rats [65].

Apoptosis/necrosis assays showed that hBN-NPs led to significant reductions in necrosis levels resulting from Aβ exposure and significantly reduced the Aβ-induced neurotoxicity. According to these results, hBN-NPs were found to have protective potential against toxicity by Aβ. In addition, IL-1β and TNF-α played key roles in astrocyte iNOS Aβ stimulation and necrosis contained TRAF6-, TRAF2- and NIK from the signaling mechanisms [66,67]. Deregulation of apoptosis was shown to play a role in the pathogenesis of various diseases involving neurodegenerative diseases, ischemic damage, autoimmune diseases and cancer. It was also thought that Aβ-induced apoptosis by causing oxidative stress or by triggering increased Fas ligand expressions in neurons and glia [68]. hBN-NPs exerted neuroprotection against Aβ-induced apoptotic and necrotic cell deaths could be attributed to anti-inflammatory action by boron content [21].

Due to the results of cytotoxicity evaluations (MTT and LDH release assays) as well as morphological analysis, we determined that cotreatment with hBN-NPs provided significant neuroprotection against Aβ. Therefore, expression levels of several genes directly or indirectly associated with Aβ metabolism were also investigated via quantitative real-time PCR. After treatment with Aβ_1-42_, the expressions of the necrosis and apoptosis pathway-related genes including BACE1, APOE, NCSTN and TNF-α genes were significantly increased while the expressions of ADAM10, BDNF, PSENEN and APH1A genes were significantly decreased. The treatment with hBN-NPs provided significant neuroprotective effects via altering expressions of these crucial AD-related genes against Aβ_1-42_ toxicity in the cellular AD model. It was found that hBN-NPs increased α-secretase activity (ADAM10) and decreased β-secretase (BACE1) which caused the formation of Aβ, the primary cause of AD. In addition, it was reported that α-, β- and γ-secretase enzymes, which play a role in the division of APP, had different effects on the formation of Aβ, and the expression and activity of these enzymes affected the level of Aβ [69]. In many studies, it was proven to be effective in the treatment of neurodegenerative diseases since the increase of ADAM10 activity, which was the main α-secretase, which broke the APP, protected the brain from the accumulation of Aβ in AD [70,71]. BACE1 was also shown to work as a β-secretase and showed all the functional features of β-secretase [72]. BACE1 inhibition by hBN-NPs might be associated with prevention of Aβ accumulation in AD.

γ-secretase regulated the amount of Aβ produced and the relative amount of 42 more toxic amino acid forms of Aβ, and it consisted of four basic subunits: presenilin (PSEN1), preseniline artifact-2 (PSEN-2), nicastrin (NCTSN) and anterior pharynx defect-1 (APH-1). Studies stated that changes in these units could increase the risk of AD [73,74,75,76]. Our findings revealed that hBN-NPs alleviated Aβ_1-42_ induced expressional alterations in *PSEN1, NCTSN* and *APH1A* genes. On the other hand, ApoE is considered to be the strongest genetic risk factor for late-onset AD [77]. It was demonstrated that fragments of ApoE, which were generated in brains of patients with AD and neuron cultures, induced NFT-like inclusions in neurofibrillary tangles (NFTs) and amyloid plaques [78]. Concordantly, the observed neuroprotective action of hBN-NPs might be also related to decreasing Aβ aggregation or increasing Aβ clearance via affecting APOE expression. In that, the elevated expression levels of APOE were suggested to contribute to the etiology of late onset AD. Thus, expressional alterations in normal protein in the brain might cause degenerative diseases [79]. On the contrary, the increased APOE expression promoted reverse transport of Aβ and led to delay of Aβ deposition in experimental mouse AD model [80].

Previous reports indicated that a reduced expression level of the BDNF gene, which played an important role in synaptic plasticity, neuronal survival, memory formation and preservation of long-term memory, was associated with neuropsychiatric and neurodegenerative disorders and plays an important role in the progression of AD [81]. Our findings indicated that hBN-NPs supported BDNF transcription that was suppressed by Aβ_1-42_ in differentiated SH-SY5Y cell cultures. Consistent with our findings, it was revealed that exposure to Aβ_1-42_ decreased the levels of BDNF transcripts IV and V in SH-SY5Y cell, and thus, BDNF was downregulated in AD [82]. Our findings also notated that hBN-NPs suppressed the expressional increases in both EGFR and TNF-α by Aβ_1-42_. Interestingly, elevated EGFR levels were well correlated with Aβ-induced memory loss in rats. In addition, inhibition of EGFR activity was introduced as an efficient treatment option for Aβ_1-42_- inclined deficits in transgenic mice [83]. Again, numerous studies have identified elevated levels of TNF-α in biological fluids in patients with aging, mild cognitive impairment, AD and epilepsy [84,85]. TNF-α induced inflammation has been reported to trigger microglia activity and neuronal death. In addition, the role of TNF-α signaling in abnormal APP processing, Aβ plaque deposition, tau-related pathology and cell death has been reported [86]. In addition, supra-physiological TNF-α was found to trigger dementia through both early and late pathogenic mechanisms [87].

## 6. Conclusions

Consequently, our study revealed the neuroprotective role of hBN- NPs against Aβ-induced neurotoxicity in SH-SY5Y cells for the first time. This investigation showed the direct and indirect anti-Alzheimer action potential of hBN-NPs. In the light of our findings, hBN-NPs were found to ameliorate neurotoxicity resulting from Aβ_1-42_ in differentiated SH-SY5Y cell culture via (I) exhibiting non-cytotoxic (<50 mg/L)/slightly toxic nature (10–50 mg/L), (II) increasing antioxidant capacity, (III) reducing Aβ-induced necrosis and apoptosis, (IV) altering mRNA expressions of responsible secretases/proteases for APP cleavage in neuron-like cells, (V) contributing to chromosome integrity, and (VI) simultaneously targeting of EGFR and TNF-α suppression and BDNF activation. Overall, hBN-NPs might be a promising and attractive core fact for brain targeted nanoformulations.

## Data Availability

The data presented in this study are available on request from the corresponding author. The data are not publicly available due to privacy.

## Supplementary Materials

The following supporting information can be downloaded at: https://www.mdpi.com/article/10.3390/nano12152690/s1, Table S1: Primer sequences for qRT-PCR.
