# Supplementary material for: Ameliorative Effects by Hexagonal Boron Nitride Nanoparticles against Beta Amyloid Induced Neurotoxicity
Source: Nanomaterials (Basel). 2022 Aug 5;12(15):2690. doi: 10.3390/nano12152690 (PMC9370266; doi:10.3390/nano12152690)
Supplement: Supplementary file 1 [file nanomaterials-12-02690-s001.zip › nanomaterials-1816922-supplementary.pdf]

nanomaterials

Article

Ameliorative Effects by Hexagonal Boron Nitride Nanoparticles against Beta Amyloid Induced Neurotoxicity

Nursah Aydin [1], Hasan Turkez [2,3], Ozlem Ozdemir Tozlu [1], Mehmet Enes Arslan [1], Mehmet Yavuz [4], Erdal Sonmez [5,6], Ozgur Fırat Ozpolat [7], Ivana Cacciatore [8], Antonio Di Stefano [8] and Adil Mardinoglu [9,10,*]

1 Department of Molecular Biology and Genetics, Erzurum Technical University, Erzurum 25050, Turkey
2 Department of Medical Biology, Faculty of Medicine, Atatürk University, Erzurum, 25240 Turkey
3 East Anatolia High Technology Application and Research Center (DAYTAM), Ataturk University, Erzurum 25240, Turkey
4 REEM Neuropsychiatry Clinics, İstanbul, 34245 Turkey
5 Department of Nanoscience and Nanoengineering, Graduate School of Natural and Applied Sciences, Ataturk University, Erzurum 25240, Turkey
6 Department of Physics, Kazım Karabekir Education Faculty, Atatürk University, Erzurum 25240, Turkey
7 Computer Sciences Research and Application Center, Atatürk University, Erzurum 25240, Turkey
8 Department of Pharmacy, University "G. d'Annunzio" of Chieti-Pescara, Via dei Vestini, 31, 66100 Chieti Scalo, CH, Italy
9 Science for Life Laboratory, KTH-Royal Institute of Technology, SE-17121 Stockholm, Sweden
10 Centre for Host-Microbiome Interactions, Faculty of Dentistry, Oral&Craniofacial Sciences, King's College London, London SE1 9RT, UK
* Correspondence: adilm@scilifelab.se

Table S1. Primer sequences for qRT-PCR.

Gene	Forward	Reverse
ADAM	AGGTGAAGAATGTGATTGTGG	GCACTGTTTCCCAGGTTTC
APH1A	CCTTTTGGGGAGTTGTGTTC	CTCATACCAGGGGTTCAGGA
APOE	GGTCGCTTTTGGGATTACCT	TTCCTCCAGTTCCGATTTGT
APP	AGTTTGTGTGTTGCCCACTG	CGTCATCATCGGCTTCTTCT
BACE1	ACTGTGCGTGCCAACATT	CGTGGGTCTGCTTTACCA
BDNF	GCCTCCTCTTCTCTTTCTGCT	TTTGTCTGCCGCCGTTAC
EGFR	CCTGCTCAACTGGTGTGTG	ATGCTGCGGTGTTTTCAC
MAPT	CCAAGACCAAGAGGGTGACA	AGCATCAGAGGTTTCAGAGC
NCSTN	CTGCTCAACGCCACTCATC	GGTAAAATGCTTGCTCTCCAG
PSENEN	CTTCTGGTTCTTCCGAGAGG	AGGAGAGGTAGTCCCCAAGG
TNF-α	CTCCTCACCCACACCATCA	GGAAGACCCCTCCCAGATAG
